# Mechanisms and applications of adipose-derived stem cell-extracellular vesicles in the inflammation of wound healing

**DOI:** 10.3389/fimmu.2023.1214757

**Published:** 2023-07-14

**Authors:** Qingyi Jia, Hanxing Zhao, Yixi Wang, Ying Cen, Zhenyu Zhang

**Affiliations:** ^1^ Department of Endocrinology and Metabolism, West China Hospital, Sichuan University, Chengdu, Sichuan, China; ^2^ Department of Plastic and Burn Surgery, West China Hospital, Sichuan University, Chengdu, Sichuan, China

**Keywords:** adipose-derived stem cells, extracellular vesicles, inflammation, wound healing, stem cell therapy

## Abstract

Wound healing is a sophisticated process consisting of serial phases with overlaps, including hemostasis, inflammation, proliferation, and remodeling. The inflammation response is an early response that plays a crucial role in eliminating microbes and clearing damaged cell debris. However, in some pathological circumstances, such as diabetes mellitus, ischemia, trauma, deep burn, etc., abnormal inflammation can cause impaired wound healing. Adipose-derived stem cells (ADSCs) belong to the mesenchymal stem cell (MSC) family and exhibit prospective applications in tissue regeneration and dermatological repairs. ADSC-secreted extracellular vesicles (ADSC-EVs) mimic the functions of ADSCs without the concerns of cell survival, immune response, or ethical issues. Studies have revealed that ADSC-EVs can inhibit abnormal inflammation responses and accelerate wound healing through various mechanisms. Moreover, some studies explored modifications in the cargo components of ADSC-EVs to enhance their therapeutic efficacy. Given the increasing studies focusing on the potential of ADSC-EVs in wound healing, how they interfere with different phases of this process has been investigated in pieces. In this review, we summarized all up-to-date evidence to map a clearer picture of the underlying mechanisms of ADSC-EVs in inflammation response. The applications of ADSC-EVs aiming at inflammation in the healing process were also reviewed to provide therapeutic strategies for future investigators.

## Introduction

The skin is the largest organ and exercises the maintenance function of balancing the external and internal environments of the human body. When the integrity of the skin is compromised, a series of repair mechanisms related to wound healing are initiated (1). Wound healing is a sophisticated and dynamic process of the body consisting of serial phases with overlaps: hemostasis, inflammation, proliferation, and remodeling (2). During the repairing process, the interplay between different cellular components of the skin is crucial and complex for proper wound healing, including fibroblasts, keratinocytes, endothelial cells, and immune cells (residential and recruited). In a physiological state, these cells and the extracellular matrix work together harmoniously to restore barrier integrity. However, factors such as metabolic diseases (insulin resistance and type 2 diabetes), prolonged mechanical stress, or vascular disorders (ischemia and vascular ulcer) can impair the healing ability, leading to chronic wounds or keloids (3).

Inflammation is one of the earliest and classic responses during wound healing. It usually occurs in the first 24 to 48 h after the injury. A moderate inflammation serves as a protective response, aiming to eliminate microorganisms and clear damaged debris, thus accelerating the healing process (4). Upon the skin injury, the coagulation cascade is activated (5). Accumulated platelets not only result in the formation of blood clots but also release various cytokines and substances, like transforming growth factor-β (TGF-β) and arachidonic acid metabolites, which recruit immune cells to the wounding site, building an immune barrier against further infection (6, 7). However, the extent of inflammation is critical, as excessive inflammation, observed in conditions such as obesity or elevated glucose levels, and deficient immune responses can lead to delayed wound healing (8). Studies also suggested that in adults, the disordered inflammatory response can drive fibrosis rather than regeneration during the healing process, potentially leading to hypertrophic scar formation (9). Since both chronic wounds and keloids are associated with reduced quality of life and increased disability rates, it highlights the significance of regulating the inflammatory phase as a potential therapeutic target in wound healing.

In the field of regeneration medicine, mesenchymal stem cells (MSCs) hold tremendous potential in tissue healing. MSCs are a group of adult cells possessing renewing capability, and they can be obtained from multiple different tissues, such as bone marrow, umbilical cord, and adipose tissue (10, 11). MSCs obtained from different sources display similar functions. Among these sources, adipose-derived stem cells (ADSCs) are often preferred as the first choice due to their accessibility and sufficient content. ADSCs can be found in subcutaneous adipose depots. They have the ability to respond to inflammation and interact with immune cells at injured sites to regulate local immune response through secreting chemokines and cytokines (12). Accumulating evidence indicates that ADSCs can release extracellular vesicles, called ADSC-EVs, through paracrine secretion to mimic ADSC behavior (13). These ADSC-EVs are considered a highly promising therapeutic strategy for restoring the balance of wound inflammation and promoting healing. Under certain pathological circumstances, administration of ADSCs directly may lead to lower survival rates due to the over-activation of inflammation. However, ADSC-EVs have resembling functions of their parental cells, while being more resistant to degradation and carrying fewer transplantation risks or ethical concerns. Therefore, ADSC-EVs present themselves as an ideal therapeutic approach for wound healing.

## Adipose-derived stem cell extracellular vesicles

Adipose tissue is the most abundant volume and one of the most functionally diverse organs in the body. In addition to its traditional storage sites functions, such as storing excessive energy into its triglyceride pool during feeding and releasing them as free fatty acids during fasting, adipose tissue is also an endocrine organ secreting adipokines, hormones, enzymes, and other bioactive particles in an endocrine, autocrine, and paracrine manner to maintain energy homeostasis. The main components of adipose tissue are mature adipocytes and the stromal vascular fraction (SVF). ADSCs are members of SVFs with self-renewal and differentiation capabilities (14). Extensive studies have proven that ADSCs not only have therapeutic effects on wounds and injuries, including cutaneous wounds, lung injuries, spinal cord injuries, etc. but have also been applied to cosmetic medicine (15, 16). In the exploration of the underlying mechanism, evidence suggested that ADSCs exert their functions through paracrine secretion of cytokines, lipids, and extracellular vesicles. These factors play crucial roles in mediating the therapeutic effects of ADSCs in different contexts.

Extracellular vesicles (EVs) represent a broad category that encompasses various types of vesicles released by living cells, carrying contents similar to those of their parental cells. EVs can be divided into exosomes, microvesicles, and apoptotic bodies based on their sizes. However, for functional purposes, EVs are commonly divided into two main groups: endosomes and exosomes (17). Exos are formed during the endosomal process, which consists of plasma membrane double invagination and the subsequent formation of multivesicular bodies (MVBs) containing multiple intraluminal vesicles (ILVs) inside (18). If the ILVs are secreted into the extracellular environment via MVB exocytosis, these ILVs are named exosomes. During this process, cytoplastic substances, including proteins, lipids, DNAs, mRNAs, and miRNAs, are encapsulated in the exosomes. Therefore, EVs are capable to conduct cell-to-cell communication without increasing the risks of rejection and malignant cell transformation (19).

Human ADSC-EVs are EVs extracted from the supernatant of ADSCs by ultracentrifugation (20). They can be identified by nanoparticle tracking analysis (NTA) and transmission electron microscopy (TEM) according to the phospholipid bilayer structure and size of ADSC-EVs (21). ADSC-EVs can also be verified by Western blot with a series of specific protein markers, such as CD9, CD63, TSG101, ALIX, and HSP90 (22, 23). As research on ADSCs continues to expand, there is a growing interest in studying ADSC-EVs. Compared to ADSC treatment, ADSC-EVs not only exhibit similar tissue repair capacities but also eliminate the concern of cellular viability, immune-mediated rejection, and malignant transformation. Thus, ADSC-EVs could achieve a novel “cell-free therapy” in regeneration medicine and wound healing.

## Inflammation in wound healing process

### The roles of immune cells in wound healing

Wound healing is a complex process that usually takes weeks or months, depending on the depth of the wound and the homeostasis of its microenvironment. Several key cell components, including immune cells, fibroblasts, and Schwann cells, play crucial roles in the wound microenvironment during the healing response. The inflammation response is one of the earliest phases in wound healing and plays a vital role in orchestrating the entire healing process. In a healthy state, neutrophils are the earliest immune cell lineages to be recruited to the site of injury. Dovi et al. demonstrated that neutrophils are responsible for killing the microorganisms at the wound site (24). After this procedure, neutrophils undergo apoptosis and are ingested by macrophages to maintain cellular homeostasis. However, in pathological conditions such as diabetic wounds, excessive neutrophil accumulation at the healing site might contribute to delayed and impaired wound healing (25).

**Figure 1:**
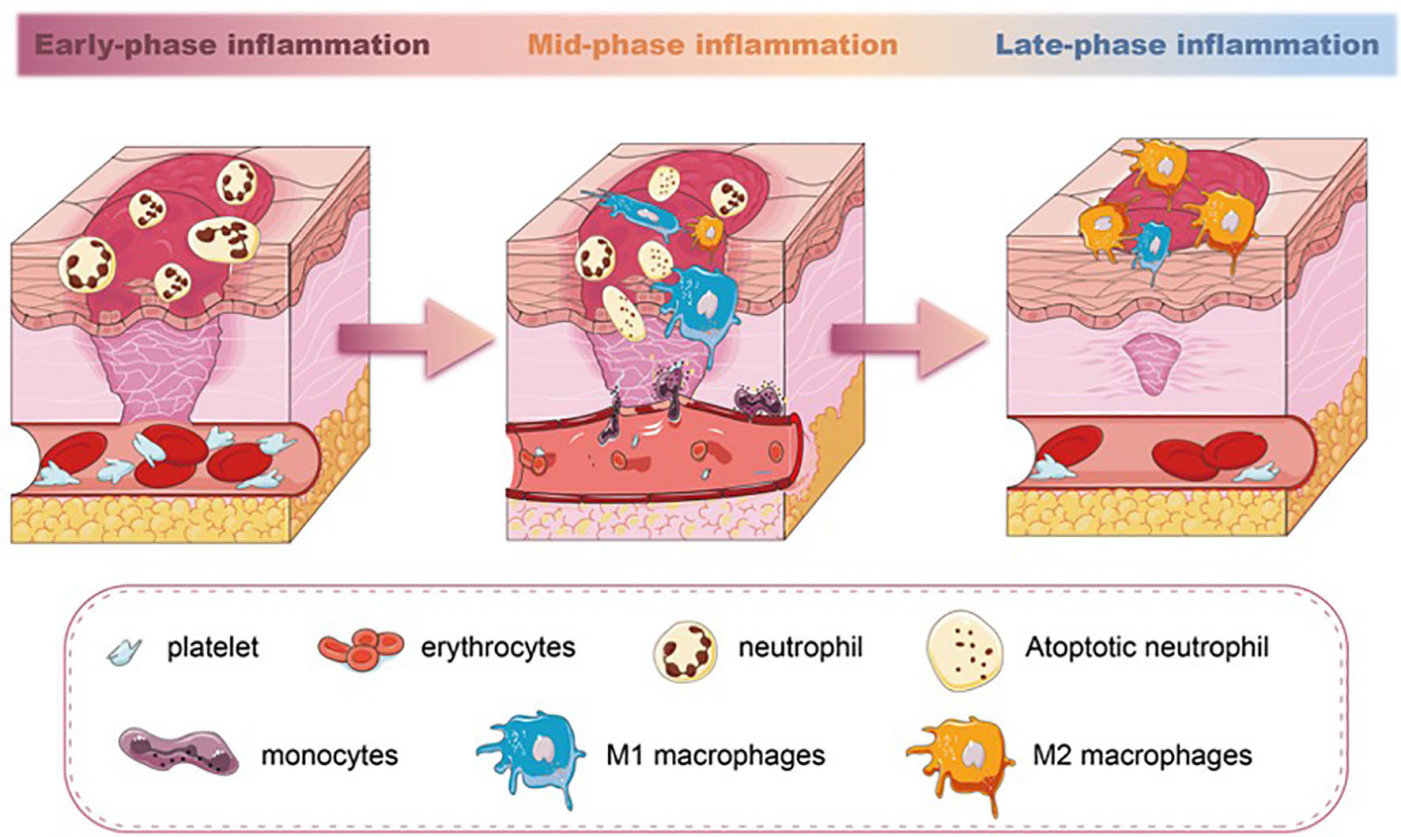
The different stages of inflammation and predominated population of immune cells in wound healing. Neutrophils are the earliest immune cell lineages during wound healing. M1 macrophages increase in the mid-phase of inflammation and phagocytose apoptotic neutrophils. In the late-phase inflammation of wound healing, a switch of M1 macrophages towards M2 macrophages is seen.

Unlike neutrophils, macrophages are essential in wound repair. The wound macrophages originate from both circulating monocytes and tissue-resident macrophage precursors. After the injury, monocytes from the circulation are rapidly recruited to the wound and simultaneously acquire macrophage phenotypic traits (26). Meanwhile, the macrophages’ precursors mature and migrate to the injured site in response to CCR2/CCL2 and CX3CR1/CXCL1 chemokines (27, 28). The number of macrophages increases, accompanied by a decrease in the number of neutrophils in the wound. This is the result of the apoptosis and phagocytosis abilities of macrophages. Once neutrophils have executed their functions, they sequentially undergo apoptosis. Macrophages recognize and engulf these neutrophils through membrane-bound tumor necrosis factor-alpha (TNF-α) and CD36 receptors (29), becoming the dominant inflammatory cell type in the wound. Studies have confirmed that the depletion of macrophages led to a lessened rate of skin wound healing (30, 31). Especially, macrophage depletion during the initial phase of injury influences the subsequent healing response, including re-epithelialization and scar formation (32). During the transition from inflammation to proliferation, the number of macrophages gradually decreases. Some of the macrophages die at the wound site, and their debris is cleared out with the wound extracellular fluid. Other macrophages migrate to the near-draining lymph nodes (33) (**Figure 1**).

Macrophages exhibit distinct phenotypes, broadly classified as proinflammatory M1 and anti-inflammatory M2 phenotypes. The two phenotypes of macrophages are able to transition from one phenotype to another depending on the microenvironment (34, 35). Each subset has a spectrum of phenotypes and their symbolic markers. Even though this dichotomous classification has been found inconclusive and arbitrary (36, 37), the markers representing different subsets can still be able to give us perspective to evaluate the inflammation severity of the tissue. During the initial stages of wound healing, both M1 macrophages and neutrophils are responsible for pathogen clearance. M1 macrophages also perform functions such as phagocytosis and the production of proinflammatory cytokines like TNF-α and interleukin-1 beta (IL-1β). As the wound progresses toward the resolution phase, M2 macrophages promote tissue remodeling, angiogenesis, and the production of anti-inflammatory cytokines, such as interleukin-10 (IL-10) and transforming growth factor-beta (TGF-β), with the highest levels occurring around day 7 of wound healing (38). M1 macrophages can exacerbate tissue damage if not regulated, and dysregulated or delayed M2 polarization can lead to chronic inflammation and impaired wound healing (39). The balance between M1 and M2 macrophages is crucial for successful wound healing. Studies have documented that the impaired switching of M1 to M2 macrophages in wounds is related to poor wound closure, impaired angiogenesis, and reduced collagen deposition (40–42). Therefore, the macrophages’ polarization during the inflammatory phase directly (phagocytosis) or indirectly (cytokines and growth factor secretion) regulates the wound-repairing process (43) (**Figure 1**).

In addition to macrophages, other immune cell lineages and their roles have also been investigated during wound healing. For example, NK cells have been found to restore the balance of antimicrobial defense in the hypoxic injury site, thereby regulating the process of skin repair (44). However, another study indicates that NK cells play a role in the delay of wound healing due to proinflammatory cytokine production (45). Further research focusing on the functions of immune cell lineages in wound healing remains an interesting and ongoing topic of investigation.

### The roles of proinflammatory and anti-inflammatory cytokines in wound healing

Numerous studies suggested that proinflammatory cytokines, including IL-1β, IL-6, and TNF-α, took part in the wound healing process, as their expression showed significantly elevated in the inflammation phase of the wound healing (46, 47). In contrast, there are also anti-inflammatory cytokines, such as IL-10, that respond to the inflammatory response. Proinflammatory cytokines are primarily produced by polymorphonuclear leukocytes and macrophages (48, 49), while anti-inflammatory cytokines are mainly produced by keratinocytes and mononuclear cells of the wound epidermis (50, 51). Despite the names of the two categories, both the appropriate levels of proinflammatory cytokines and the coordinated expression of anti-inflammatory cytokines are crucial for normal wound healing.

TNF-α is a potent proinflammatory cytokine released early in wound healing. It activates endothelial cells, promotes leukocyte recruitment, and induces the production of other cytokines and chemokines, thereby initiating the inflammatory response (52). Knockdown of TNF-α receptor p55-mediated signals has been shown to positively affect wound healing and reduce leukocyte infiltration (53). IL-6, secreted by various cell types, including macrophages and fibroblasts, plays a role in the early stages of wound healing. It stimulates immune cell activation, angiogenesis, and fibroblast proliferation, contributing to tissue repair (54). Gallucci et al. demonstrated that IL-6 deficiency in mice resulted in impaired cutaneous wound healing and a single dose of recombinant IL-6 was able to reverse the situation (55). IL-1β, predominantly produced by macrophages, promotes the recruitment of immune cells, angiogenesis, and the production of matrix metalloproteinases (MMPs) (56). On the other hand, the anti-inflammatory cytokine IL-10 is a potent anti-inflammatory cytokine produced by immune cells such as macrophages and T cells. It suppresses proinflammatory cytokine production, dampens immune cell activation, and promotes tissue repair and regeneration (57). Studies have demonstrated that fetal cells heal the wound scarlessly, while the absence of IL-10 in fetal cells resulted in scar formation (58). Also, overexpression of IL-10 in adult mice led to decreased inflammation response and wound healing acceleration (59). Taken together, proinflammatory and anti-inflammatory cytokines play distinct yet interconnected roles in wound healing. Proinflammatory cytokines initiate the inflammatory response and recruit immune cells, while anti-inflammatory cytokines resolve inflammation and promote tissue repair and remodeling. Achieving a balanced cytokine response is critical for effective wound healing.

## Inflammatory effects of ADSC-EVs in wound healing

A moderate and appropriate inflammation in the wounding site is critical and a footstone for the whole healing course, as we have mentioned. Studies using different inflammatory markers all showed ADSC-EVs have the ability to alleviate the intensity of inflammation response at the injury wound site. In normal and healthy individuals, tissue undergoing wound healing showed significantly decreased infiltration of inflammatory cells when treated with ADSC-EVs (60–62). Furthermore, ADSC-EV administration has been found to substantially downregulate the expression of proinflammation cytokines IL-6 and TNF-α while upregulating anti-inflammation cytokine levels of IL-10 (62, 63). Zhou et al. showed that ADSC-EVs treatment either intravenously or by smearing on the wounding site can both remarkably reduce macrophage CD68 and M1-macrophage CD14 in the skin lesion (64). Similarly, Heo et al. observed a significant increase in the expression of the M2-macrophage marker CD206 in cells treated with ADSC-EVs (65). The polarization of macrophages toward an anti-inflammatory M2 phenotype promoted by ADSC-EVs suppresses the release of proinflammatory cytokines such as TNF-α and IL-1β while enhancing the secretion of anti-inflammatory cytokines such as IL-10, leading to a shift in the macrophage phenotype toward tissue repair and regeneration (66).

In pathological conditions, like diabetic individuals, the effectiveness of ADSC-EVs in ameliorating the inflammation response becomes more prominent. ADSC-EVs can significantly decrease IL-6, TNF-α, and IL-1β expression (67–70), suppress CD14 and CD68 expression levels (68), and remarkably increase CD206 and IL-10 expression levels (69, 71) in diabetic wound lesions. Histological analysis has also indicated reduced immune cell infiltration, thus preventing the formation of diabetic ulcers (72, 73). In addition to the local effects, ADSC-EVs can reverse the systematic inflammatory condition in diabetes models. Jian et al. found ADSC-EV treatment can significantly decrease serum IL-6, IL-1β, and TNF-α levels (74). Other diseases that are not specifically for wound healing but are involved in similar phases of wound repairment, such as ischemic injury, nerve injury, and epithelial recovery, showed that ADSC-EVs can substantially modulate the balance of inflammation response. Studies revealed that ADSC-EVs impact a number of inflammatory pathways and cytokines in brain and nerve injuries (75, 76). Fewer neutrophils and macrophages infiltrated the fistula after ADSC-EV administration (77), and ADSC-EVs have been shown to modulate neutrophil function by reducing neutrophil activation, oxidative stress, and release of proinflammatory mediators. This modulation helps limit excessive inflammation and tissue damage at the wound site (78), increasing the number of CD206 counted to aid urethral defect recovery after using ADSC-EVs (79). Cao et al. also demonstrated that ADSC-EVs can significantly decrease the number of CD11b-positive macrophages and resolve inflammation after microneedle-induced injury (80).

## How ADSC-EVs regulate wound inflammation

There is no doubt that ADSC-EVs can alleviate wound inflammation response. However, the mechanisms underneath this phenomenon are heat topics being discussed. EVs consist of three main substances: RNAs, proteins, and lipids (81). RNAs are the most widely investigated, as exosomes contain a spectrum of RNAs that conduct intercellular communication (**Table 1**).

**Table 1:** Regulation mechanism of ADSC-EVs in wound and injury inflammation.

Mechanism	Subtypes	Models	Phenotype/pathway	Ref.
Micro-RNA	miR-34a-5p, miR-124-3p, and miR-146-5p	Fibroblast scratch model	IL-6↓, TNF-a↓, IL-8↓, IL-10↑, TSG-6↑, TGF-β1↑; M2 macrophage polarization↑	(65)
	miR-132 and miR-146a	THP-1 cells treated with LPS; a full-thickness skin wound of diabetic nude mice	ROCK1/PTEN signaling pathway↓; M2 macrophage polarization↑	(82, 83)
	miR-21-5p	A full-thickness skin wound of a murine model; a full-thickness excision wound of a diabetic rat	M2 macrophage polarization↑, KLF6↓	(63, 84)
	miR-21-3p, miR-126-5p, and miR-31-5p↑; miR-99b, miR-146-a↓	A full-thickness skin wound of diabetic nude mice	PI3K/AKT signaling pathway↑	(70)
	miR-146a	HUVECs; CD14+ monocytes	IL-1β↓; M2 macrophage polarization↑; NF-κB signaling mediators IRAK1	(85, 86)
	miR-29a	A scald skin wound of mice model	Inflammatory cells infiltration↓	(87)
	miR-10a	Peritonitis in C57BL/6 mice	M1 macrophage polarization↓, NF-κB signaling pathway↓	(88, 89)
	miR-30d-5p	Acute ischemic stroke of rat *in vivo* model and oxygen- and glucose-deprived (OGD) primary microglia *in vitro* model	M2 macrophage polarization↑; IL-6↓, TNF-a↓, iNOS↓; IL-10↑, IL-4↑	(75)
	miR-146a-5p, miR-340-5p, miR-223-3p, miR-125b-5p, miR-16-5p, miR-149-3p, miR-105-5p, miR-181c-3p, miR-146b-5p, and miR-181a-5p	LPS-treated THP-1 cells	Selenium; IL-6↓, TNF-a↓, IL-8↓; IL-10↑, TGF-β1↑, CD206↑, CD163↑, Arg1↑	(90)
	miR-511-3p	Rats with spinal cord injury; PC12 cells under LPS damage	TRAF6/S1P/NF-κB signaling pathway↓	(91)
linc-RNA	lncRNA H19	A full-thickness skin wound of a mouse model	miR-19b↑, SOX9↑, Wnt/β-catenin signaling pathway↑	(61)
	lncRNA GAS5	LPS-treated HDF cells	TLR7 signaling pathway↓	(92)
	linc00511	Rat diabetic foot model	IL-6↓, TNF-a↓, IL-1β↓	(74)
	lncGm37494	*In vitro* and *in vivo* spinal cord injury	miR-130b-3p↓, IL-6↓, TNF-a↓, IL-1β↓; PPARγ↑, M2 macrophage polarization↑	(93)
circ-RNA	circ-Snhg11	A full-thickness skin wound of diabetic mice	miR-144-3p↓, STAT3 signaling pathway↓; M2 macrophage polarization↑	(71)
	mmu_circ_0000250	A full-thickness skin wound of diabetic mice	miR-128-3p↓; SIRT1↑	(72)
	circ-Fryl	A sepsis-induced lung injury mouse model and an LPS-induced alveolar epithelium cells damage model	miR-490-3p↓; SIRT3/AMPK↑	(94)
Immunomodulatory characteristics	M2 macrophage polarization	Chondrocytes and synoviocytes	IL-1β↓, p65 nuclear translocation↓, NF-κB signaling pathway↓	(95)
	Neutrophil function	Rat diabetic foot model	Interferon-γ↓, M1↓, ROS↓, Nrf2↑; IL-6↓, TNF-a↓, IL-1β↓; inflammatory cell infiltration↓; EGR-1↑	(67, 96, 97)
	Treg-cell activation	A full-thickness skin wound of a mouse model	Interferon-γ↓, M1 macrophage polarization↓; EFGR↑	(98)
	Reduced B cell proliferation	Peripheral blood mononuclear cell	Proliferation↓, differentiation↓	(99)
	Reduced inflammatory cytokines	HUVECs under high glucose; a full-thickness skin wound of db/db mice model	ROS reduced through SIRT3	(100)
	Vimentin	A full-thickness skin wound of a mice model; human dermal fibroblasts	IL-6↓, TNF-a↓; IL-10↑	(62)

### ADSC-EV RNAs modulate wound inflammation

RNAs are commonly divided into three types: messenger RNA (mRNA), transfer RNA (tRNA), and ribosomal RNA (rRNA). These three types of RNAs carry out almost all cellular regulatory processes. RNAs can also be divided into coding RNA (cRNA) and noncoding RNA (ncRNA). The ncRNA can be subdivided into long ncRNAs (lncRNAs) and microRNAs (miRNAs) according to their size. Circular RNAs (circRNAs) are unique from other RNAs due to their 5′ and 3′ ends bonding together and creating a loop (101). Studies confirmed that lncRNAs, miRNAs, and circRNAs are all able to regulate cell physiological functions and realize intercellular communication (102). Recent research found ADSC-EVs to be a vital source of ncRNAs to engage in the inflammation response in wound healing (103). Heo et al. reported that miR-34a-5p, miR-124-3p, and miR-146-5p expressed in ADSC-EVs attenuated IL-6 expression and induced M2-phenotype macrophage polarization in the fibroblast scratch model (65). Further investigation suggested that miR-132 and miR-146a improved the anti-inflammatory responses through ROCK1 and PTEN signaling pathways in THP-1 cells (82). Especially, miR-132 from ADSC-EVs is found to induce M2 polarization in diabetic skin flaps (83). Li et al. observed that ADSC-EV treatment of diabetic foot ulcer wounds could elevate miR-21-5p levels in macrophages, induce M2 polarization, and suppress Keuppel-like factor 6 (KLF6), which has been reported to enhance the inflammatory phenotype in macrophages (63, 84). Wang et al. indicated that miR-21-3p, miR-126-5p, and miR-31-5p upregulation and miR-99b and miR-146-a downregulation in hypoxic ADSC-EVs regulated immune response via phosphatidylinositide 3-kinases (PI3K)/protein kinase B (AKT) signaling pathway, thus accelerating wound healing in the diabetic model (70). Water et al. studied the miR-146a in the inflammatory responses exhibited by endothelial cells. miR-146a secreted by ADSC-EVs is capable of inhibiting inflammatory activation by IL-1β (85), and miR-146 might participant in M2 polarization by targeting NF-κB signaling mediators IRAK1 (86). Yuan et al. reported that miR-29a overexpression in ADSC-EVs could reduce inflammatory cell infiltration and subsequentially forbid keloid formation after skin burn (87). Baglio et al. screened the top 5 miRNAs in ADSC-EVs using small RNA sequencing and found miR-486-5p, miR-10a-5p, miR-10b-5p, miR-191-5p, and miR-222-3p accounted for almost half of the miRNAs (88). Later, Njock et al. confirmed that among these five miRNAs, miR-10a was able to suppress M1 activation by targeting the NF-κB signaling pathway in peritonitis (89). Treatment with ADSC-EVs overexpressed with miR-30d-5p showed a positive effect on M2 polarization, decreased expression of TNF-α, IL-6, and iNOS, and increased IL-4 and IL-10 levels after acute stroke (75). In the spinal cord injury model, hypoxia-pretreated ADSC-EVs enriched miR-511-3p and ameliorated the inflammation response via the TRAF6/S1P/NF-κB pathway (91). Despite the fact that overexpressing miRNAs in the ADSC-EVs can directly verify their roles in enhancing ADSC-EV function in inflammatory regulation of wound healing, Heo et al. found that pretreatment of selenium in ADSC during culture could also improve the inflammatory cytokines through miRNAs. Selenium-treated ADSC-EVs significantly suppressed the inflammatory response in THP-1 cells due to the elevated miRNAs of miR-146a-5p, miR-340-5p, miR-223-3p, miR-125b-5p, miR-16-5p, miR-149-3p, miR-105-5p, miR-181c-3p, miR-146b-5p, and miR-181a-5p (90).

The circRNAs attenuating inflammation reaction in wounding lesions is supported by circ-Snhg11. Hypoxic treatment significantly increased circ-Snhg11 contents in ADSC-EVs and promoted M2 polarization by inhibiting miR-144-3p expression and the STAT3 signaling pathway in skin wounds (71). The mmu_circ_0000250 in ADSC-EVs was verified to promote miR-128-3p absorption, which can induce inflammatory response, and subsequently increase SIRT1 level and improve hyperglycemic-induced inflamed environment in diabetic wound sites (72). Shen et al. studied the overexpression of circ-Fryl in ADSC-EVs, regulating miR-490-3p to attenuate inflammation-related injury via SIRT3/AMPK pathway (94). Qian et al. studied lncRNA H19 in ADSC-EVs targeting miR-19b and SOX9 to activate the Wnt/β-catenin pathway, thus promoting wound healing and alleviating inflammation responses in skin wounds (61). lncRNA GAS5 in ADSC-EVs was found to also decrease inflammatory response in human dermal fibroblasts (HDF) by downregulating the TLR7 signaling pathway (92). Qiu et al. found that linc00511 overexpression in ADSC-EVs decreased serum IL-6, IL-1β, and TNF-α levels in the diabetic foot model (74). ADSC-EVs overexpressed with lncGm37494 downregulated its downstream target miR-130b-3p and subsequently upregulated PPARγ expression, which is a miR-130b-3p target, shifting M2 polarization and downregulating TNF-α, IL-6, and IL-1β (93).

### ADSC-EVs modulate immunomodulatory characteristics

**Figure 2:**
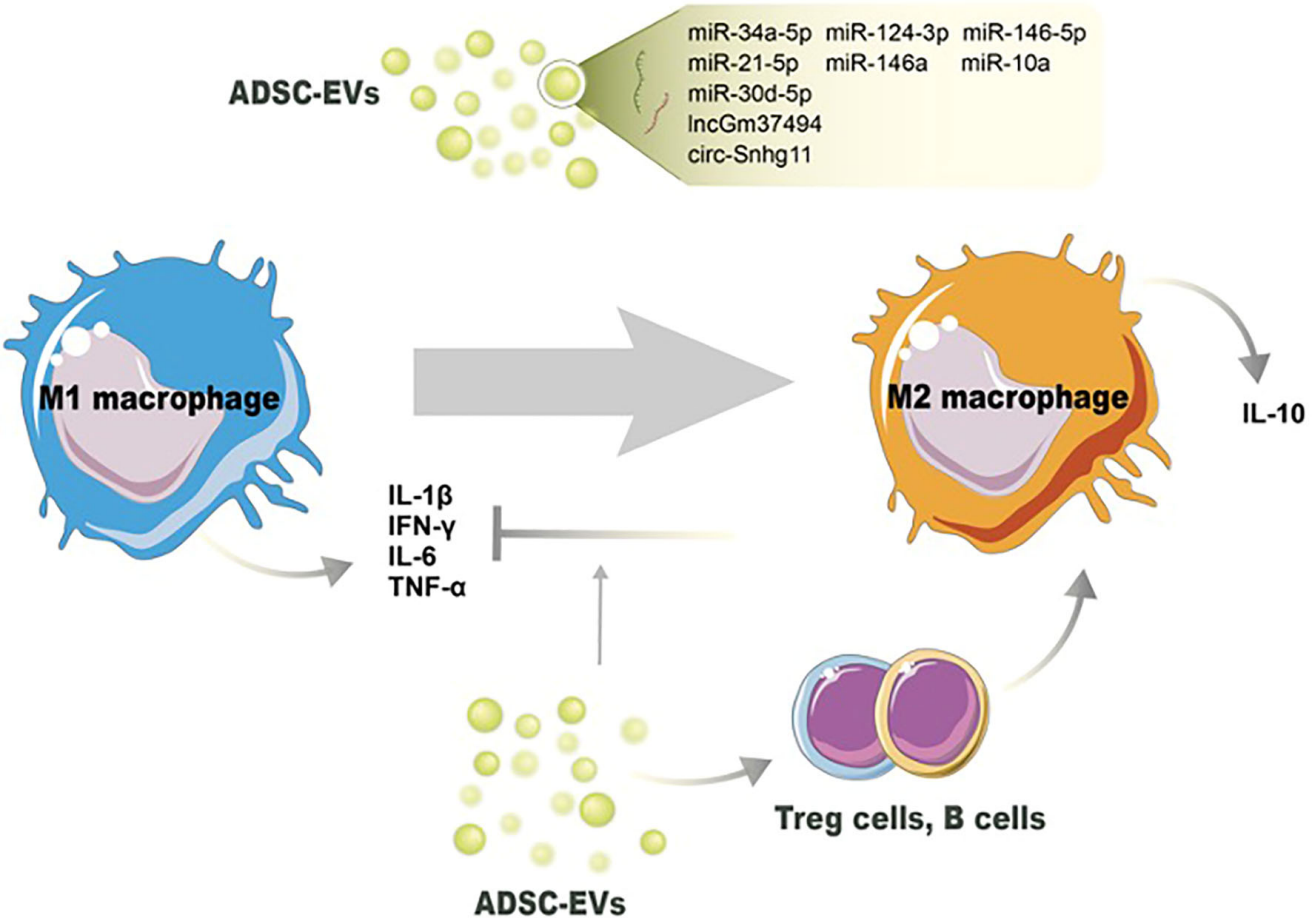
Potential mechanisms of ADSC-EVs regulating the switch of M1 macrophages toward M2 macrophages. Noncoding RNAs in ADSC-EVs induce M2 macrophage polarization and decrease the number of M1 macrophages. ADSC-EVs also modulate other immune cells’ activation to switch macrophage phenotype and reduce proinflammation cytokines production.

The immunomodulatory characteristics of ADSC-EVs are presented by the influence of immune cell activities and non-ncRNA-related macrophage polarization. ADSC-EVs have been shown to influence macrophage polarization, shifting the balance from a proinflammatory M1 phenotype towards an anti-inflammatory M2 phenotype, indicating the anti-inflammation action (104). The underpinning mechanism, besides the changes of miRNA contents in ADSC-EVs, might be due to the changed proinflammatory cytokine levels in the microenvironment. Proinflammatory cytokines, including IL-1β, IL-6, and TNF-α, can classically activate the M1 phenotype, and suppressing them can reverse M1/M2 switching (105). Cavallo et al. confirmed the mechanism by which ADSC-EVs reverse the proinflammatory microenvironment and induce the expression of anti-inflammation cytokines. After pretreated with IL-1β, ADSC-EV administration could prevent p65 nuclear translocation and NF-κB signaling pathway activation. The decreased expression of IL-1β, IL-6, and TNF-α was consistent with the blockage of the proinflammatory pathway. On the other hand, ADSC-EVs significantly increased IL-10 and IL-4 levels, inducing an alternative action of M2 macrophages (95) (**Figure 2**).

ADSC-EVs have been found to modulate neutrophil function, leading to the suppression of their activation and subsequent reduction in proinflammatory responses. Neutrophils generate reactive oxygen species (ROS) as part of their antimicrobial defense mechanism. However, excessive ROS production can cause tissue damage and impair wound healing. ADSC-EVs have been shown to reduce oxidative stress in neutrophils by regulating the production of ROS and restoring redox balance (96). Li et al. proved that overexpression of Nrf2 in ADSC-EVs decreased the detoxification effect of ROS under hyperglycemic conditions and proinflammatory cytokines IL-1β, IL-6, and TNF-α levels (67). As we mentioned above, excessive or uncontrolled neutrophil infiltration can prolong inflammation and impair wound healing. ADSC-EVs have been shown to modulate neutrophil migration by influencing the expression of adhesion molecules and chemotactic factors. This regulation helps maintain the appropriate balance of neutrophils at the wound site, preventing excessive tissue damage. Sun et al. demonstrated that ADSC-EVs influenced inflammatory cell infiltration through the expression of early growth response factor-1 (EGR-1) (97). A study employed by Parvanian et al. reported that vimentin-knockout ADSC-EVs showed an extended inflammation phase in sounds and failed to recruit inflammatory cells to the wound site. The absence of vimentin in ADSC-EVs upregulated proinflammatory cytokines IL-6 and TNF-α, and downregulated anti-inflammatory cytokine IL-10 (62). In conclusion, ADSC-EVs exhibit a variety of mechanisms by which they suppress neutrophil activation and modulate inflammation during wound healing.

ADSC-EVs also possess other immunomodulatory properties and can suppress the activation and function of various immune cells involved in wound healing. Nosbausm et al. showed that ADSC-EVs promoted T-regulatory cell activation and facilitated wound healing by inhibiting interferon-γ production and M1 macrophage accumulation in an EFGR signal-dependent manner (98). B cells are involved in antibody production and can contribute to inflammation in certain contexts (106). ADSC-EVs have been reported to modulate B-cell responses, leading to reduced B-cell proliferation and antibody production (99). Zhang et al. found that ADSC-Exos significantly decreased inflammatory cytokines IL-6, TNF-α, and MCP-1 levels by reducing ROS production and protecting mitochondrial function through SIRT3 (100). All these immune modulations help maintain an appropriate balance of immune cells and inflammation during wound healing. Taken together, ADSC-EVs have emerged as potent regulators of wound inflammation through various mechanisms. These include the modulation of macrophage polarization, suppression of neutrophil infiltration, and immune cell suppression. Understanding the intricate mechanisms underlying ADSC-EV-mediated regulation of wound inflammation will pave the way for the development of targeted therapies and improved wound healing outcomes. Further research is needed to uncover additional mechanisms and optimize the use of ADSC-EVs as therapeutic interventions in the context of wound healing.

## Application of ADSC-EVs

The therapeutic use of ADSC-EVs shows a promising future as a “cell-free-therapy” approach in wound healing. Nevertheless, ADSC-EVs usually lead to rapid clearance due to the high metabolic activity of wounds. Therefore, investigators keep making an attempt to combine biotechnology and ADSC-EVs that enable optimized retention and release profiles of ADSC-EVs. Hyaluronic acid (HA) and hydrogels were used as exosome immobilizers as well as ideal wound dressing. By combining HA and ADSC-EVs, a distinct decreased in inflammatory cell infiltration was observed in the wound area (107). Hydrogels are three-dimensional networks that can encapsulate and deliver ADSC-EVs to the wound site. They provide a supportive environment for cell growth, facilitate the sustained release of ADSC-EVs, and enhance their therapeutic effects. ADSC-EV-loaded hydrogels have been shown to alleviate inflammation, promote tissue regeneration, and improve wound healing outcomes. Some hydrogels with modifications are capable of alleviating inflammation responses in wound healing themselves. Silva et al. investigated a thermoresponsive gel-embedded ADSC-EV formulation. The gel itself could significantly reduce neutrophil and macrophage infiltration in the necrotic area, while combining gel and ADSC-EVs could further decrease the immune cell burden (77). Zhou et al. developed a thermosensitive hydrogel, Pluronic F-127, and found this material not only reduced IL-6 level in the cutaneous wound but also significantly reduced inflammation (decreased IL-6, TNF-α, CD68, and increased CD206) when encapsulated ADSC-Exos (108). We also developed a methacryloyl-modified gelatin (GelMA) hydrogel with catechol motifs of dopamine (GelMA-DOPA) and loaded it with ADSC-EVs to treat diabetic wounds. The GelMA-DOPA can relieve IL-6 expression in lesions, and GelMA-DOPA-EVs lower the inflammation burden more efficiently (109).

Another material serving as a structural framework for cell attachment and tissue regeneration is scaffolding. Incorporating ADSC-EVs into scaffolds can enhance their regenerative capacity and immunomodulatory effects. ADSC-EV-loaded scaffolds have been demonstrated to reduce inflammation, enhance angiogenesis, and promote wound closure. A study showed that a 3D scaffold engineered from decellularized cardiac tissue was an ideal support material for ADSC-EVs to employ anti-inflammatory functions in ischemic myocardial infarction (110). A more biocompatible scaffold of the human acellular amniotic membrane (hAAM) revealed effective inflammation regulation functions. Xiao et al. found that hAAM loaded with ADSC-EVs could enhance inflammatory regulation in diabetic wounds (111). Similarly, nanofibers are also considered a desirable material for ADSC-EV bedding due to their high surface area-to-volume ratio, mimicking the extracellular matrix structure. The research found that phosphoethanolamine phospholipid-grafted poly-l-lactic acid micro/nanofibers (DSPE-PLLA) not only could carry ADSC-EVs and release them in a slow manner, but also showed M2 macrophage polarization with increased expression of Arginase1, CD206, and IL-10 (112). These data present strong evidence for the prospective future of ADSC-EVs in alleviating inflammation responses and promoting wound healing. The association of different biomaterials and ADSC-EVs cannot only preserve their activities and function but also extend their release time, showing promising clinical pharmacotherapy for wound healing. However, blinded, randomized, placebo-controlled, and a larger number of prospective clinical trials need to be carried out to verify the safety and effectiveness of ADSC-EVs in the future.

## Conclusion

A considerable number of clinical patients are suffering from prolonged wound healing due to an imbalanced inflammation response, especially under pathological conditions. Inappropriate immune responses in injury lesions affect the whole healing process. Despite the number of studies focusing on the efficacy of ADSC-EV mechanism of alleviating the inflammatory response to promote wound healing, modified applications targeting these mechanistic factors are still vacant. ADSC-EVs obtain the analogous functions of ADSCs and act as a carrier to conduct an action on target cells at the transcription and translation level to transmit proteins, messenger RNA, microRNAs, circ-RNAs, lincRNAs, and cytokines via paracrine manners. Based on these biological characteristics, ADSC-EVs modulate and control the cytokines production, macrophage phenotype polarization, and immune cell lineage infiltration through their contents. Furthermore, with the combination of newly developed bioengineered materials and media, the effect of ADSC-EVs controlling immunomodulation can be persistent and even enhanced in some types of materials. It is undeniable that the significance of ADSC-EVs’ effects has presented a new opportunity for wound healing in clinical practice. Nevertheless, further drug development focusing on the targeted mechanism pathway of ADSC-EVs *in vivo* and *in vitro* is needed to expand our clinical options, and clinical trials of ADSC-EVs are needed for wound healing.

## Author contributions

QJ and ZZ conceptualized and validated the manuscript. QJ drafted the manuscript. YW, HZ, YC, and ZZ reviewed and edited the manuscript. QJ and HZ drew the figures in the manuscript. QJ and ZZ obtained the funding. ZZ supervised the project. QJ and ZZ are the guarantors of this work and, as such, had full access to all the data in the study and take responsibility for the integrity of the data and the accuracy of the data analysis. All authors have read and agreed to the published version of the manuscript, accepted responsibility for the entire content of this manuscript, and approved its submission.
